# Dynamic Trend of Myocardial Edema in Takotsubo Syndrome: A Serial Cardiac Magnetic Resonance Study

**DOI:** 10.3390/jcm11040987

**Published:** 2022-02-14

**Authors:** Ken Kato, Michiko Daimon, Masanori Sano, Koki Matsuno, Yoshiaki Sakai, Iwao Ishibashi, Tadayuki Kadohira, Koji Matsumoto, Yoshitada Masuda, Takashi Uno, Jelena-Rima Ghadri, Christian Templin, Yoshio Kobayashi

**Affiliations:** 1 Department of Cardiovascular Medicine, Graduate School of Medicine, Chiba University, Chiba 260-8670, Japan; miko0122@kkd.biglobe.ne.jp (M.D.); yoshio.kobayashi@wonder.ocn.ne.jp (Y.K.); 2 Department of Cardiology, Chiba Emergency Medical Center, Chiba 261-0012, Japan; m.sano@mac.com (M.S.); kmatsuno323@yahoo.co.jp (K.M.); yo-sak@gf6.so-net.ne.jp (Y.S.); 3 Department of Cardiology, Akimoto Hospital, Chiba 273-0121, Japan; rocky12qq@aol.com; 4 Department of Cardiology, Tokyo Metropolitan Police Hospital, Tokyo 164-8541, Japan; t-kadohira@mua.biglobe.ne.jp; 5 Department of Radiology, Chiba University Hospital, Chiba 260-8677, Japan; matumoto@chiba-u.jp (K.M.); masuda.yoshitada@hospital.chiba-u.jp (Y.M.); unotakas@faculty.chiba-u.jp (T.U.); 6 Department of Cardiology, University Heart Center, University Hospital Zurich, 8091 Zurich, Switzerland; jelena-rima.templin-ghadri@usz.ch (J.-R.G.); christian.templin@usz.ch (C.T.)

**Keywords:** takotsubo syndrome, takotsubo cardiomyopathy, cardiovascular magnetic resonance, myocardial edema, transient apical wall thickening

## Abstract

Background: The wall motion abnormalities of the left ventricle (LV) in takotsubo syndrome (TTS) are known to be transient and completely recover within a few weeks. However, there is little information about the relationship between functional recovery and tissue characteristics. The aim of this study was to investigate the recovery process of TTS using cardiovascular magnetic resonance (CMR). Methods: Consecutive patients with TTS were prospectively enrolled. We performed serial CMR in the acute phase (<72 h after admission), the subacute phase (7–10 days after admission) and the chronic phase (3 months later). To assess the degree of myocardial edema quantitatively, we evaluated the signal intensity of myocardium on T2-weighted images and calculated the signal intensity ratio compared with the skeletal muscle. Results: Fifteen patients with TTS were enrolled. CMR demonstrated reduced LV ejection fraction in the acute phase, and it recovered almost completely by the subacute phase. On the other hand, severe myocardial edema was still observed in the subacute phase, associated with increased LV mass. The highest signal intensity ratio in the subacute phase was correlated with the maximum voltage of negative T wave on electrocardiogram (r = 0.57, *p* = 0.03). Conclusions: In patients with TTS, myocardial edema associated with increased LV mass still remained in the subacute phase despite functional recovery of the LV. Electrocardiogram may be useful to assess the degree of myocardial edema in the subacute phase. Our study suggests that myocardial ischemia might have a central role in developing TTS.

## 1. Introduction

Takotsubo syndrome (TTS) is characterized by transient systolic dysfunction of the left ventricle (LV) and has been increasingly recognized since its first description in 1990 in Japan [1]. In general, the wall motion abnormality is known to completely recover within a few weeks [2], but there is limited knowledge about the detailed recovery process. Recently, echocardiographic studies demonstrated that transient apical wall thickening (TAWT) seems similar to apical hypertrophic cardiomyopathy, observed in the subacute phase during the recovery process of TTS, and has been associated with worse in-hospital outcome [3]. However, pathological information on TAWT could not be obtained by echocardiography.

Cardiovascular magnetic resonance (CMR) has been used often in clinical studies regarding TTS because of its benefit in providing both functional information and tissue characterization of myocardium [4]. Previous studies on TTS using CMR demonstrated that transient myocardial edema was observed in the area of wall motion abnormality in the acute phase on T2-weighted images [5,6]. Although it was also reported that myocardial edema was not shown in the chronic phase, there is no study performing CMR in the subacute phase that assesses the pathological change during the recovery process. In addition, the presence of late gadolinium enhancement (LGE) in TTS has been widely debated and remains controversial [5,7,8,9].

Thus, the aim of the present study was to explore the changes of LV systolic function and myocardial tissue characteristics by serial CMR during the recovery process in TTS patients.

## 2. Methods

### 2.1. Study Population

A total of 15 consecutive TTS patients without contraindication of CMR who were admitted to Chiba University Hospital and Chiba Emergency Medical Center from October 2013 to March 2016 were enrolled. TTS was diagnosed according to the following criteria [10,11]: (1) transient regional wall motion abnormalities of the LV which extended beyond a single epicardial vascular distribution; (2) absence of obstructive coronary artery disease or angiographic evidence of acute plaque rupture; (3) new electrocardiographic abnormalities or modest elevation in cardiac troponin; and (4) absence of pheochromocytoma or myocarditis. Coronary angiography and left ventriculography were performed in all patients during the acute phase, and the absence of obstructive coronary artery disease, explaining the LV contraction abnormality, was confirmed. All images were reviewed by 2 investigators blind to the clinical information. Disagreements were resolved by consensus. We performed serial CMR in the acute phase (<72 h after admission, N = 15), the subacute phase (7–10 days after admission, N = 14) and the chronic phase (3 months later, N = 13). Electrocardiogram and blood sample analysis were performed on admission and at the times of CMR. All participants provided written informed consent. The ethical committee of Chiba University and Chiba Emergency Medical Center approved the study.

### 2.2. Cardiovascular Magnetic Resonance

CMR was performed using a standardized protocol on 1.5-T magnetic resonance scanner with a 5-channel phased-array coil (in Chiba University Hospital: Achieva, Philips Medical Systems, Best, The Netherlands; in Chiba Emergency Medical Center: Avanto, Siemens Medical Solutions, Erlangen, Germany). Imaging consisted of cine (steady-state free precession images), T2-weighted (short-tau inversion recovery images) and LGE images (3-dimensional [Achieva, Philips Medical Systems] or 2-dimensional [Avanto, Siemens Medical Solutions] inversion recovery gradient echo images), and we obtained a LV 4-chamber view, a 2-chamber view, and a stack of short-axis views in each series during breath-holds gated to the electrocardiogram. Short-axis views were acquired from the atrioventricular ring through the LV at intervals of 8 mm thickness with 0 to 2 mm gap in cine images and 8–10 mm thickness with −5 to 2 mm gap in LGE images, and by three slices (at basal, mid and apex area) in T2-weighted images. The LGE images were acquired 10 to 15 min after intravenous administration of a gadolinium-based contrast agent (Magnevist, Schering AG, Berlin, Germany, or Meglumine Gadopentetate, Fuji Pharma, Toyama, Japan; 0.15 mmol/kg) in patients without contraindications of LGE.

### 2.3. Image Analysis

Offline analysis was performed using commercially available software (Virtual Place Advance, Aze, Tokyo, Japan). LV functional analysis was performed by tracing endocardial and epicardial borders in short-axis cine CMR images throughout the cardiac cycle to calculate LV volume, ejection fraction, and LV mass. For quantitative assessment of myocardial edema, we traced myocardium manually with caution to exclude bright artifacts due to slow blood flow on basal, mid, and apical slices of short-axis T2-weighted images. We evaluated the mean signal intensity of myocardium on respective slices and compared that with the signal intensity of skeletal muscle on the same slices to calculate the signal intensity ratio (SIR) [5,12]. The highest SIR out of 3 slices was defined as SIR_max_. For assessment of LGE, a region of interest was selected in an apparently normal myocardium in the 4-chamber images, and automated detection to 5 standard deviations above the mean signal intensity of normal myocardium was performed to identify fibrosis [5]. All measurements were performed by a blind, experienced CMR investigator.

### 2.4. Statistical Analysis

Continuous variables are listed as mean ± standard deviation (SD) or as median and interquartile range (IQR). Categorical variables are presented as counts and percentages. One-way factorial analysis of variance, repeated measures analysis of variance, or Friedman test were performed for comparisons of multiple variables as appropriate. Post hoc analysis was performed using Tukey’s test or paired *t*-test/Wilcoxon signed-rank test with Bonferroni correction as appropriate. Statistical analysis was performed using JMP Pro 13 (SAS Institute, Cary, NC, USA). A *p* value of <0.05 was considered statistically significant.

## 3. Results

### 3.1. Baseline Patient Characteristics

Baseline characteristics are presented in Table 1. All patients were female, and the mean age was 71. Mean LV ejection fraction by left ventriculography was 48%. Two-thirds of patients showed apical ballooning and others were atypical variants. Serial laboratory and electrocardiographic findings were summarized in Table S1. White blood cell count decreased and corrected QT interval shortened significantly from the acute to the subacute phase.

### 3.2. CMR Imaging

**Figure 1:**
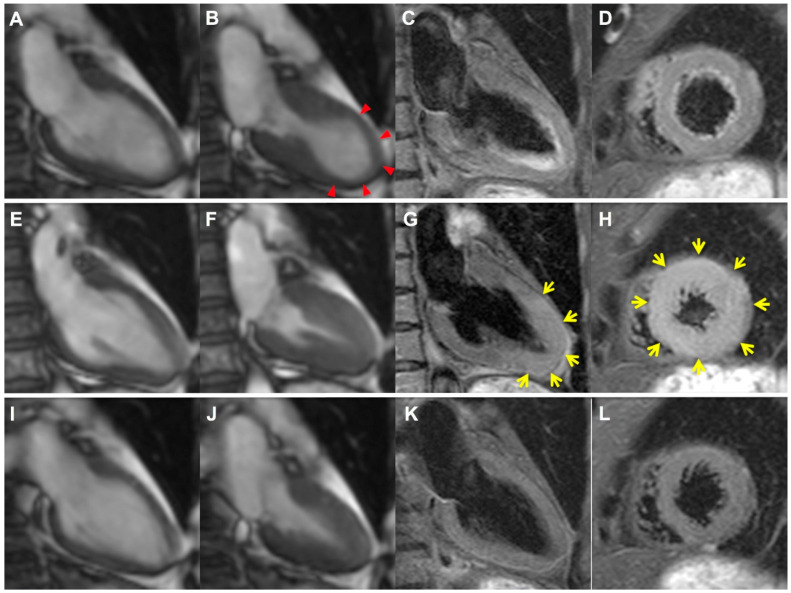
Serial change of myocardial edema in a representative patient with TTS. In the acute phase, apical ballooning was observed ((**A**,**B**), red arrows). Myocardial edema on T2-weighted images was slight (**C**,**D**). In the subacute phase, wall motion was completely recovered (**E**,**F**), whereas more thickened apical wall with more severe myocardial edema was observed compared with the acute phase ((**G**,**H**), yellow arrows). In the chronic phase, the final CMR imaging demonstrated normal systolic function (**I**,**J**) without apical wall thickening or myocardial edema (**K**,**L**). CMR, cardiovascular magnetic resonance; TTS, takotsubo syndrome. Adapted with permission from ref. [13]. Copyright 2017 the Japanese Circulation Society.

**Figure 2:**
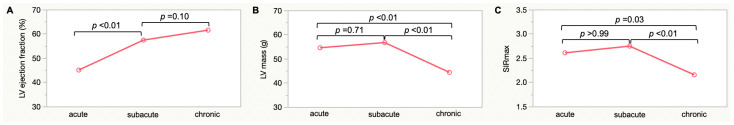
Serial Change of LV ejection fraction, LV mass, and myocardial edema. Reduced LV ejection fraction was observed in the acute phase, and it recovered almost completely by the subacute phase (**A**). On the other hand, the most severe myocardial edema with increased LV mass was shown in the subacute phase, and both of them decreased in the chronic phase (**B**,**C**). LV, left ventricular; SIR, signal intensity ratio.

CMR was performed in the acute phase (median Day 2, IQR 1–2 days), the subacute phase (median Day 8, IQR 7–9), and the chronic phase (median Day 89, IQR 83–91). Serial CMR findings were summarized in Table 2 and representative images were shown in Figure 1. CMR in the acute phase demonstrated reduced LV ejection fraction that recovered almost completely by the subacute phase (42 ± 13 vs. 56 ± 10 vs. 62 ± 6%, *p* < 0.01, Figure 2A). On the other hand, no change in LV mass was observed until the subacute phase. After that, it decreased significantly (53 ± 16 vs. 56 ± 16 vs. 44 ± 14 g, *p* < 0.01, Figure 2B).

Increased SIR_max_ in the acute phase was maintained in the subacute phase despite recovery of systolic function. After that, it decreased significantly in the chronic phase in parallel with LV mass reduction (Table 2, Figure 2C). SIR_max_ in the subacute phase was correlated with the maximum amplitude of negative T wave on electrocardiogram performed at the same time (r = 0.57, *p* = 0.03).

The LGE images were obtained only from patients with normal renal function at each time. Myocardial fibrosis was identified in 21% (3/14) of patients in the acute phase, 25% (3/12) in the subacute phase, and 42% (5/12) in the chronic phase. All detected LGE showed patchy distribution, while transmural or subendocardial LGE, often observed in myocardial infarction or in myocarditis respectively, was not detected in this study cohort. Patients who had LGE in the chronic phase tended to show higher SIR_max_ in the subacute phase (3.1 ± 0.5 vs. 2.5 ± 0.6, *p* = 0.14).

## 4. Discussion

The main results of this study were: (1) LV systolic function recovered almost completely by the subacute phase; (2) myocardial edema and increased LV mass remained in the subacute phase despite recovery of systolic function; (3) both were resolved in the chronic phase. To the best of our knowledge, this is the first prospective study to evaluate changes of LV systolic function and tissue characterization during the recovery process of TTS using a serial CMR three times.

Previous studies reported that wall motion abnormality recovered completely within a few weeks in almost all TTS cases [2,14]. However, there is limited knowledge regarding the relationship between functional recovery and tissue characterization. Ahtarovski et al. reported that systolic function recovered almost completely before discharge, which is consistent with our result [15]. Recent reports demonstrated that TAWT, which mimics apical hypertrophic cardiomyopathy, was temporarily observed by echocardiography in the subacute phase of TTS, and subsequently resolved at follow-up [3]. Our result in regards to LV mass, which increased from the acute to the subacute phase, can strengthen the results of previous echocardiographic studies. In addition, we evaluated myocardial edema serially. In the acute phase, SIR_max_ had already been higher than the normal range, and increased SIR_max_ remained in the subacute phase despite recovery of systolic function. After that, it was depressed significantly in the chronic phase. These trends of myocardial edema during the recovery process were similar to those of LV mass, which suggests that the cause of TAWT is myocardial edema.

Shin et al. reported that TAWT was associated with worse in-hospital outcome [3]. Thus, serial evaluation of myocardial edema may be useful to follow TTS patients. However, in general, it is difficult to perform serial CMR in a clinical setting. We demonstrated the correlation between the degree of myocardial edema and the maximum amplitude of negative T wave on electrocardiogram in the subacute phase. This observation is consistent with the previous study showing the correlation between myocardial edema and negative T wave [16]. Thus, serial electrocardiogram may be helpful to manage TTS patients, providing additional information of myocardial edema.

From the pathophysiological perspective, it is of great interest why severe myocardial edema was shown in the subacute phase even after recovery of systolic function. The definitive pathophysiology of TTS is still unclear, although several hypotheses, such as multivessel coronary spasm, catecholamine-mediated myocardial stunning, excessive transient ventricular afterload, and microcirculatory dysfunction have been postulated [17]. Furthermore, myocardial edema is recognized as one of the possible etiologies of TTS, because myocardial edema was often observed in the acute phase with severe wall motion abnormality in previous studies [5]. However, the results of the present study may suggest that myocardial edema is not a direct etiology of TTS but a secondary phenomenon after other pathophysiological causes. Fernandez-Jimenez et al. demonstrated in their recent study enrolling patients with myocardial infarction that an initial reperfusion-related wave of myocardial edema appears immediately after reperfusion, and is followed by a second healing-related wave of edema peaking around 7 days after reperfusion [18]. In the present study, we revealed that severe myocardial edema was shown around 8 days after admission despite functional recovery of the LV in TTS. This phenomenon is consistent with the edematous reaction after myocardial infarction, suggesting myocardial ischemia might play a key role in the pathophysiology of TTS.

Although the absence of LGE was classically considered as one of the typical features of TTS, recent studies revealed that LGE was observed in TTS patients. Eitel et al. demonstrated that focal or patchy LGE was observed in 9% of TTS patients in the acute phase [5]. However, the exact pathophysiology of LGE in TTS is still unclear. Rolf et al. reported that 5 of 15 TTS patients showed LGE in the acute phase, which was due to the increase in the extracellular matrix, and the LGE was completely reversed in the chronic phase [7]. On the other hand, Naruse et al. found patchy LGE remained in 3 of 8 TTS patients at 6-month follow-up [8]. They also demonstrated that patients with LGE at follow-up showed higher peak creatine kinase levels compared with those without LGE. Based on these results, they speculated that the cause of LGE in the chronic phase might be myocardial contraction-band necrosis, which can explain a patchy, not transmural, distribution of LGE. This hypothesis is consistent with previous histological analyses of TTS [19,20]. In addition, a previous speckle tracking echocardiographic study demonstrated residual impairment in the apical segment at long-term follow-up [21]. In the present study, 5 of 12 patients had LGE in the chronic phase and tended to show more severe myocardial edema in the subacute phase. This result can be interpreted as secondary myocardial edema in the subacute phase may be related to minute irreversible myocardial damage, which does not affect global systolic function. However, it may be associated with a long-term outcome, and a further study with a large number of patients will be needed.

### Study Limitations

Several limitations of this study should be acknowledged. First, the long-term outcomes of this study’s cohort were not assessed. The sample size was too small to evaluate association between CMR findings and long-term prognosis. Second, atypical variants of TTS were included in this study. However, this limitation did not affect our results because we could assess functional and pathological findings of the whole heart in detail using CMR. Third, we could not perform an endomyocardial biopsy to confirm histological findings because of its invasive nature.

## 5. Conclusions

In patients with TTS, the wall motion abnormality of the LV recovers almost completely by the subacute phase, whereas myocardial edema associated with increased LV mass still remains despite functional recovery of the LV. The degree of myocardial edema is correlated with the maximum amplitude of negative T wave on electrocardiogram in the subacute phase. Thus, serial electrocardiogram may be useful as surrogate information for myocardial edema. In addition, our study provides important insights into the pathophysiology of TTS, suggesting myocardial ischemia might have a central role in developing TTS. To confirm our hypothesis, further studies with a large number of patients will be needed.

## Figures and Tables

**Table 1:** Baseline Clinical Characteristics.

Variable	All
	(*n* = 15)
Age, years	71 ± 5
Female	15 (100%)
BMI, kg/m^2^	22.5 ± 3.3
Coronary risk factors	
Hypertension	9 (60%)
Dyslipidemia	7 (47%)
Diabetes mellitus	3 (20%)
Smoking	1 (7%)
Symptoms	
Chest pain	10 (67%)
Dyspnea	2 (13%)
Triggers	
Emotional stress	6 (40%)
Physical stress	3 (20%)
No apparent trigger	6 (40%)
ECG findings at presentation	
ST elevation	9 (60%)
T wave inversion	12 (80%)
QTc, msec	493 ± 72
Troponin elevation	14 (93%)
Maximal CK myocardial band, U/L	18.0 (11.0–22.9)
LV ejection fraction (LVG), %	48 ± 12
Ballooning pattern	
Apical ballooning	10 (66%)
Atypical variants	5 (33%)

Values are shown as mean ± SD, median (IQR), or *n* (%); BMI = body mass index; CK = creatine kinase; ECG = electrocardiography; QTc = corrected QT interval; LV = left ventricular; LVG = left ventriculography.

**Table 2:** Serial findings of cardiovascular magnetic resonance.

	Acute	Subacute	Chronic	*p* Value
LV ejection fraction, %	42 ± 13	56 ± 10	62 ± 6	<0.01
LVEDV, mL	93 ± 20	88 ± 19	88 ± 21	0.20
LVESV, mL	54 ± 17	39 ± 12	34 ± 11	0.01
LV stroke volume, mL	39 ± 14	49 ± 13	54 ± 13	<0.01
LV mass, g	53 ± 16	56 ± 16	44 ± 14	<0.01
SIR_max_	2.7 ± 0.6	2.8 ± 0.6	2.2 ± 0.4	<0.01
LGE, *n*/total *n* (%)	3/14 (21)	3/12 (25)	5/12 (42)	0.56

Values are shown as mean ± SD, or *n*/total *n* (%). LGE = late gadolinium enhancement; LV = left ventricular; LVEDV = LV end-diastolic volume; LVESV = LV end-systolic volume; SIR = signal intensity ratio.

## Data Availability

The data presented in this study are available on request from the corresponding author.

## Supplementary Materials

The following supporting information can be downloaded at: https://www.mdpi.com/article/10.3390/jcm11040987/s1, Table S1: Serial findings of laboratory and electrocardiographic data.
