# Supplementary material for: Dynamic Trend of Myocardial Edema in Takotsubo Syndrome: A Serial Cardiac Magnetic Resonance Study
Source: J Clin Med. 2022 Feb 14;11(4):987. doi: 10.3390/jcm11040987 (PMC8878106; doi:10.3390/jcm11040987)
Supplement: Supplementary file 1 [file jcm-11-00987-s001.zip › jcm-1574310-supplementary.pdf]

Table S1. Serial findings of laboratory and electrocardiographic data.

	Acute	Subacute	Chronic	P value
Laboratory data				
WBC (/μl)	10000 (6100-12300)	5600 (4000-7400)	6500 (5250-7700)	<0.01
CRP (mg/dl)	0.22 (0.04-0.73)	0.11 (0.02-0.79)	0.04 (0.02-0.12)	0.06
BNP (pg/ml)	328 (155-500)	104 (68-140)	45 (30-59)	0.09
ECG findings				
HR (bpm)	72 ± 13	64 ± 10	69 ± 11	0.01
QTc (msec)	504 ± 72	442 ± 32	426 ± 24	0.01

BNP = brain natriuretic peptide; CRP = C-reactive protein; ECG = electrocardiography; HR = heart rate; QTc = corrected QT interval; WBC = white blood cell count.
